# Exploration of daily Internet data traffic generated in a smart university campus

**DOI:** 10.1016/j.dib.2018.07.039

**Published:** 2018-07-27

**Authors:** Oluwaseun J. Adeyemi, Segun I. Popoola, Aderemi A. Atayero, David G. Afolayan, Mobolaji Ariyo, Emmanuel Adetiba

**Affiliations:** a Center for Systems and Information Services, Covenant University, Ota, Nigeria; b Department of Electrical and Information Engineering, Covenant University, Ota, Nigeria; c HRA, Institute for Systems Science, Durban University of Technology, Durban, South Africa

**Keywords:** Smart campus, Internet Protocol, Internet data traffic, Nigerian university, Smart education

## Abstract

In this data article, a robust data exploration is performed on daily Internet data traffic generated in a smart university campus for a period of twelve consecutive (12) months (January–December, 2017). For each day of the one-year study period, Internet data download traffic and Internet data upload traffic at Covenant University, Nigeria were monitored and properly logged using required application software namely: FreeRADIUS; Radius Manager Web application; and Mikrotik Hotspot Manager. A comprehensive dataset with detailed information is provided as supplementary material to this data article for easy research utility and validation. For each month, descriptive statistics of daily Internet data download traffic and daily Internet data upload traffic are presented in tables. Boxplot representations and time series plots are provided to show the trends of data download and upload traffic volume within the smart campus throughout the 12-month period. Frequency distributions of the dataset are illustrated using histograms. In addition, correlation and regression analyses are performed and the results are presented using a scatter plot. Probability Density Functions (PDFs) and Cumulative Distribution Functions (CDFs) of the dataset are also computed. Furthermore, Analysis of Variance (ANOVA) and multiple post-hoc tests are conducted to understand the statistical difference(s) in the Internet traffic volume, if any, across the 12-month period. The robust data exploration provided in this data article will help Internet Service Providers (ISPs) and network administrators in smart campuses to develop empirical model for optimal Quality of Service (QoS), Internet traffic forecasting, and budgeting.

**Specifications Table**

**Table:** 

Subject area	*Engineering*
More specific subject area	*Information and Communication Engineering*
Type of data	*Tables, graphs, figures, and spreadsheet file*
How data was acquired	*For each day of the one-year study period, Internet data download traffic and Internet data upload traffic at Covenant University, Nigeria were monitored and properly logged using an open source software, FreeRADIUS, Radius Manager web application, and Mikrotik Hotspot Manager.*
Data format	*Raw, analyzed*
Experimental factors	*Internet data download traffic and Internet data upload traffic were monitored and logged for only nineteen (19) days in December, 2017 because the university proceeded to end-of-year break afterward.*
Experimental features	*Descriptive statistics, boxplot representations, time series plots, frequency distributions, correlation and regression analyses, Probability Density Functions (PDFs), Cumulative Distribution Functions (CDFs), Analysis of Variance (ANOVA) test, and multiple post-hoc test are performed to explore the dataset provided in this data article. All statistical computations were done using the Machine Learning and Statistics toolbox in MATLAB 2016a software.*
Data source location	*The dataset on Internet data traffic provided in this article were collected at Covenant University, Canaanland, Ota, Nigeria (Latitude 6.6718*° *N, Longitude 3.1581*° *E)*
Data accessibility	*A comprehensive dataset is provided in Microsoft Excel spreadsheet file and attached as*supplementary material*to this data article for easy research utility and validation*

**Value of the data**

• The data provided in this data article can be used to accurately predict Internet data traffic in a smart campus environment. Predictions of Internet data traffic will help network engineers to improve the Quality of Service (QoS) of computer networks and also ensure efficient utilization of the networks in a smart university campus [1], [2].
• Availability of dataset on Internet data traffic obtained from real scenarios will facilitate more empirical research in the areas of computer networking and Internet traffic engineering [3], [4].
• This dataset is made available to give correct facts and figures on Internet data traffic in a Nigerian university campus that is driven by Information and Communication Technologies (ICTs) [5], [6].
• Free access to daily Internet data traffic of a period of one year will facilitate the development of empirical prediction models that can be used by Internet Service Providers (ISPs) and Internet subscribers in a smart university campus for effective network planning and traffic forecasting [7], [8], [9], [10], [11], [12].
• Robust data exploration that is performed in this data article will help the university network administrators to gain useful insights about the traffic peak and off-peak periods. Also, the descriptive statistics, frequency and probability distribution plots, correlation analysis, ANOVA test and multiple post-hoc test results will give better understanding of the relationships between the Internet data download traffic and the Internet data upload traffic in a smart campus [13], [14], [15].

## 1 Data

Ubiquitous access to reliable Internet services is pivotal to achieving sustainable smart education in university campuses [16], [17], [18]. Accurate Internet data traffic prediction models are required for computer network planning and forecasting to guarantee efficient Quality of Service (QoS) in enterprise computer networks and applications. However, computer network planning are usually carried out based on theoretical formulations and simulations due to paucity of empirical data from real life scenarios. In this data article, a robust data exploration is performed on daily Internet data traffic in a smart university campus for a period of twelve consecutive (12) months (January–December, 2017).

For each month, descriptive statistics of daily Internet data download traffic and daily Internet data upload traffic are presented in tables. The mean, median, mode, standard deviation, variance, kurtosis, Skewness, range, minimum, maximum, and sum of the daily Internet data traffic download and upload for January–December, 2017 are presented in Tables 1 and 2 respectively.

**Table 1:** Descriptive statistics of daily IP data download traffic in Terabytes (TB).

	**Jan**	**Feb**	**Mar**	**Apr**	**May**	**Jun**	**Jul**	**Aug**	**Sep**	**Oct**	**Nov**	**Dec**
Mean	2.28	2.30	2.88	2.72	2.41	2.23	1.89	1.20	3.15	3.20	3.17	2.33
Median	2.60	2.40	2.90	2.60	2.20	2.20	1.90	0.92	3.25	3.20	3.00	2.40
Mode	3.40	2.00	2.90	2.50	2.00	2.00	2.10	0.82	3.50	3.00	3.00	2.40
Standard Deviation	1.21	0.66	0.79	0.52	0.82	0.49	0.71	0.80	0.40	0.49	0.69	0.97
Variance	1.46	0.44	0.63	0.27	0.66	0.24	0.51	0.64	0.16	0.24	0.48	0.94
Kurtosis	1.61	4.38	5.12	3.80	3.18	3.67	5.58	6.03	2.63	1.78	5.56	3.96
Skewness	−0.47	−1.16	0.48	−0.16	0.95	0.81	0.87	2.00	−0.69	0.11	0.76	0.75
Range	3.51	2.74	4.20	2.60	3.30	2.00	3.70	3.30	1.50	1.60	3.80	4.09
Minimum	0.19	0.36	1.20	1.30	1.20	1.50	0.60	0.50	2.20	2.40	1.40	0.81
Maximum	3.70	3.10	5.40	3.90	4.50	3.50	4.30	3.80	3.70	4.00	5.20	4.90
Sum	70.81	64.36	89.40	81.60	74.80	67.00	58.46	37.20	94.40	99.10	95.00	44.21

**Table 2:** Descriptive statistics of daily IP data upload traffic in Terabytes (TB).

	**Jan**	**Feb**	**Mar**	**Apr**	**May**	**Jun**	**Jul**	**Aug**	**Sep**	**Oct**	**Nov**	**Dec**
Mean	0.29	0.43	0.49	0.48	0.36	0.31	0.28	0.19	0.58	0.65	0.64	0.33
Median	0.32	0.47	0.47	0.50	0.29	0.31	0.29	0.15	0.59	0.64	0.65	0.28
Mode	0.14	0.07	0.15	0.14	0.14	0.20	0.06	0.06	0.40	0.69	0.23	0.14
Standard Deviation	0.16	0.14	0.14	0.09	0.18	0.06	0.11	0.14	0.06	0.06	0.12	0.17
Variance	0.02	0.02	0.02	0.01	0.03	0.00	0.01	0.02	0.00	0.00	0.02	0.03
Kurtosis	1.71	3.55	5.43	8.61	3.49	2.45	2.55	5.07	4.47	2.89	7.23	6.52
Skewness	−0.24	−1.04	0.19	−2.07	1.19	0.18	−0.07	1.80	−1.16	0.16	−0.39	1.74
Range	0.51	0.56	0.73	0.47	0.66	0.25	0.48	0.52	0.27	0.28	0.77	0.72
Minimum	0.03	0.07	0.15	0.14	0.14	0.20	0.06	0.06	0.40	0.53	0.23	0.14
Maximum	0.54	0.63	0.88	0.60	0.80	0.44	0.54	0.58	0.67	0.80	1.00	0.86
Sum	8.96	12.17	15.12	14.27	11.26	9.23	8.66	5.94	17.29	20.10	19.06	6.24

## 2 Experimental design, materials and methods

**Fig. 1:**
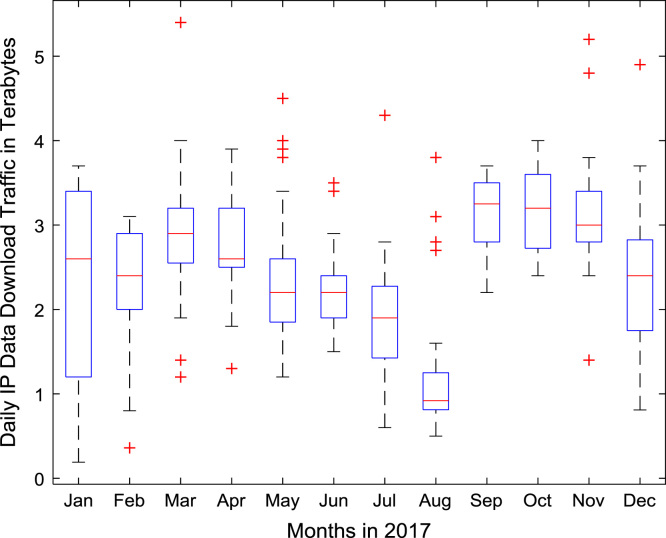
Boxplot representation of daily data download traffic in Terabytes (TB).

**Fig. 2:**
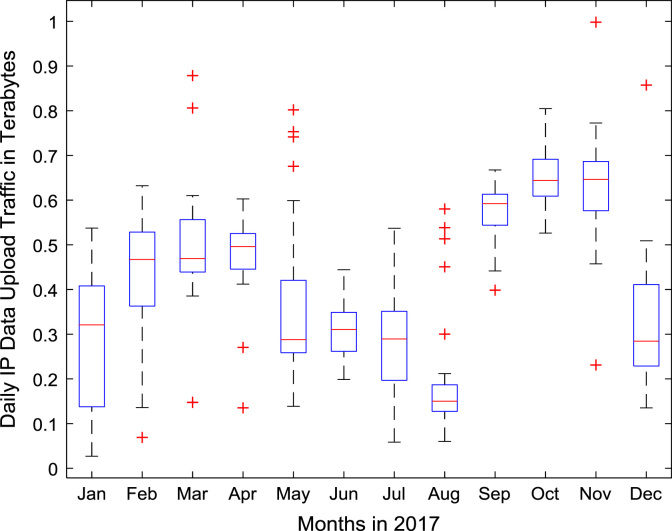
Boxplot representation of daily data upload traffic in Terabytes (TB).

A robust data exploration was performed on daily Internet data traffic in a smart university campus for a period of twelve consecutive (12) months (January–December, 2017). For each day of the one-year study period, Internet data download traffic and Internet data upload traffic at Covenant University, Nigeria were monitored and properly logged using an open source software, FreeRADIUS, Radius Manager web application, and Mikrotik Hotspot Manager. FreeRADIUS software was installed in Linux Operating System (OS) for authentication, authorization, and accounting services. Radius Manager Web application was used to add users, to edit and create cards, and to harvest data in a more user-friendly format. Mikrotik Hotspot Manager was used to integrate the smart campus network to the enterprise edge. Statistical computations were done using the Machine Learning and Statistics toolbox in MATLAB 2016a software. Boxplot representations of the daily download traffic and the daily upload traffic for the 12-month period are shown in Figs. 1 and 2 respectively.

## 3 Data exploration

**Fig. 3:**
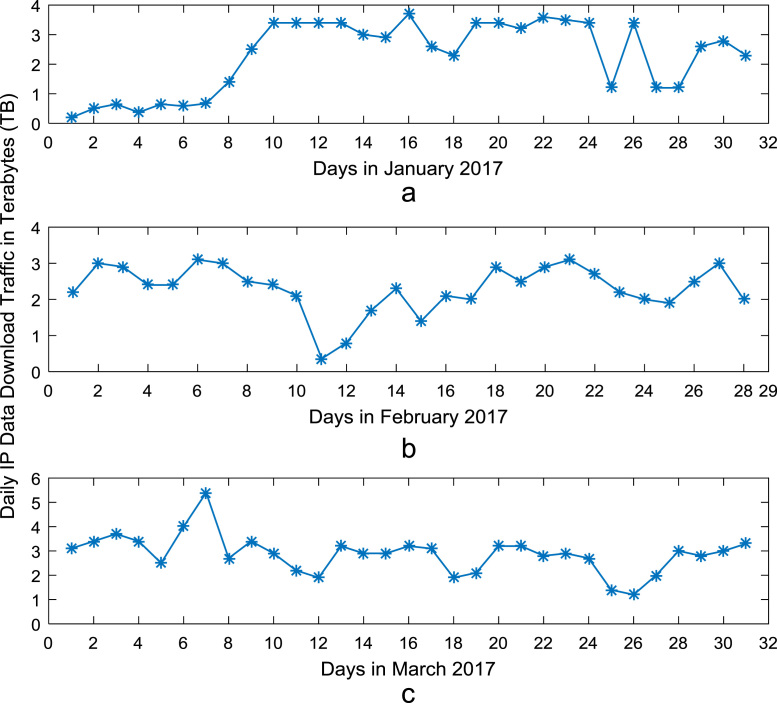
(a)–(c). Download traffic volume in first quarter, 2017.

**Fig. 4:**
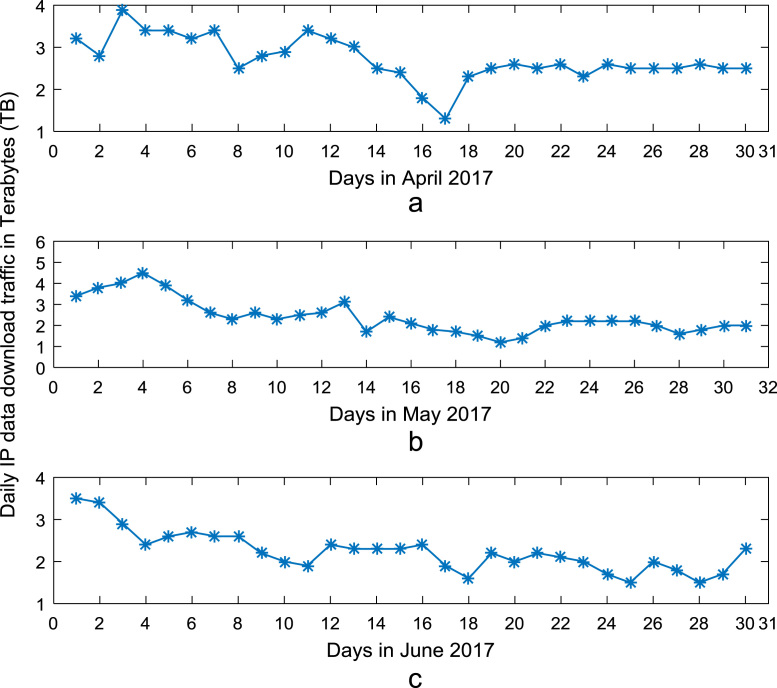
(a)–(c). Download traffic volume in second quarter, 2017.

**Fig. 5:**
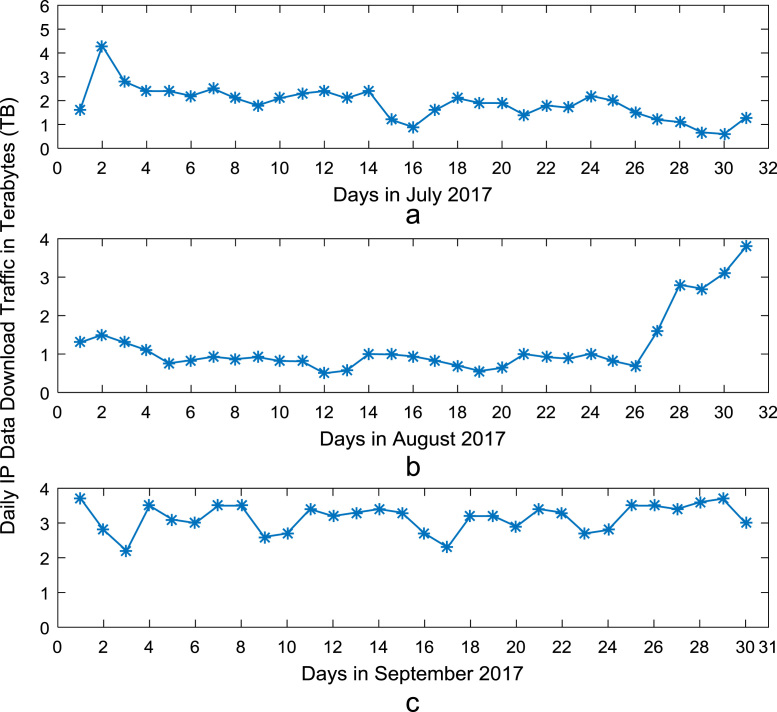
(a)–(c). Download traffic volume in third quarter, 2017.

**Fig. 6:**
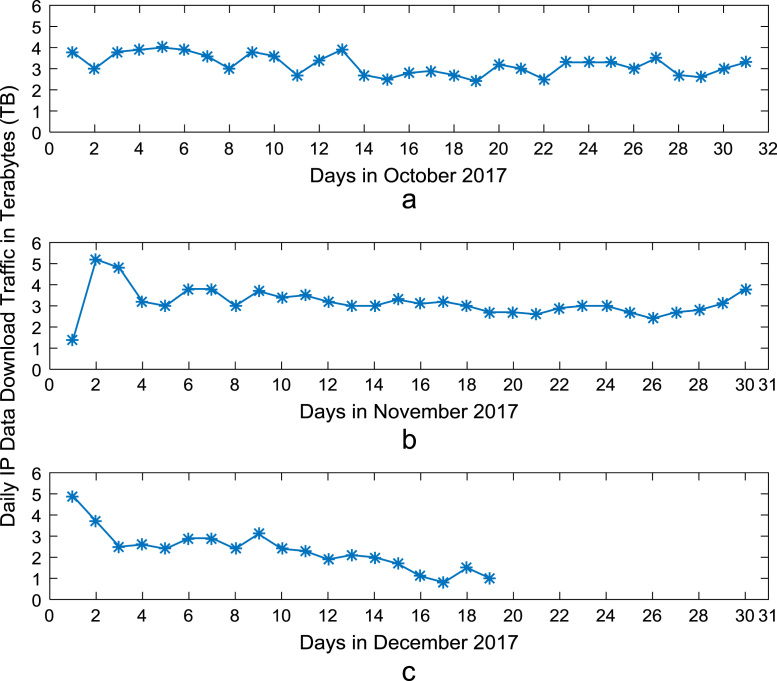
(a)–(c). Download traffic volume in fourth quarter, 2017.

**Fig. 7:**
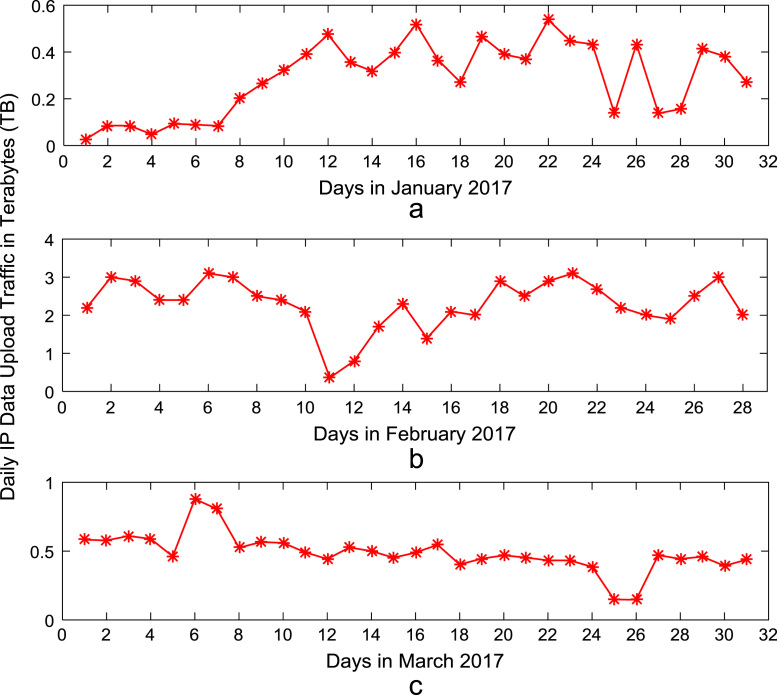
(a)–(c). Upload traffic volume in first quarter, 2017.

**Fig. 8:**
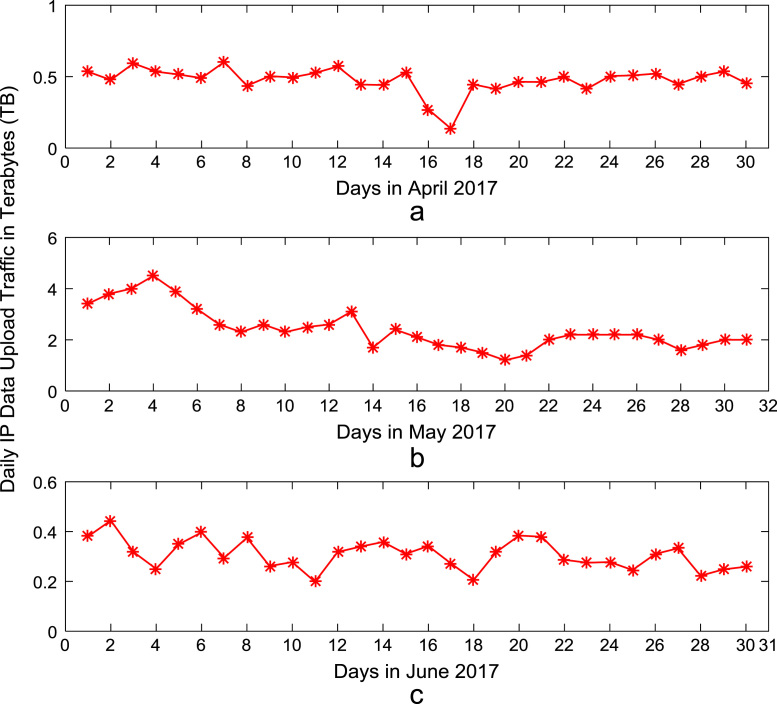
(a)–(c). Upload traffic volume in second quarter, 2017.

**Fig. 9:**
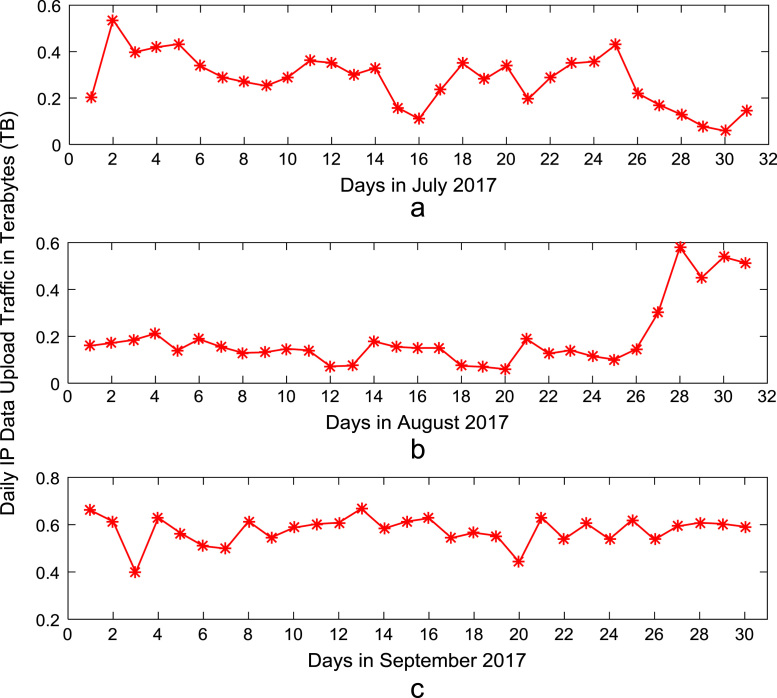
(a)–(c). Upload traffic volume in third quarter, 2017.

**Fig. 10:**
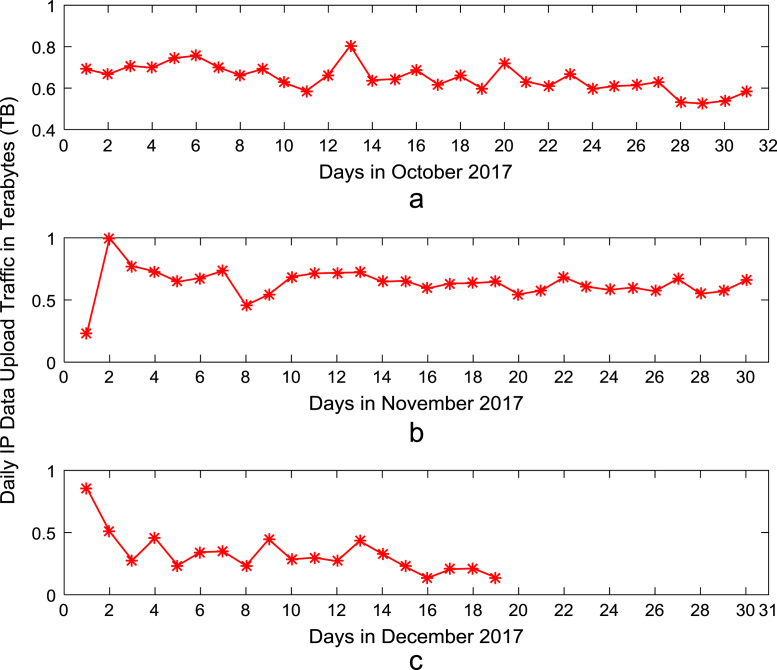
(a)–(c). Upload traffic volume in fourth quarter, 2017.

**Fig. 11:**
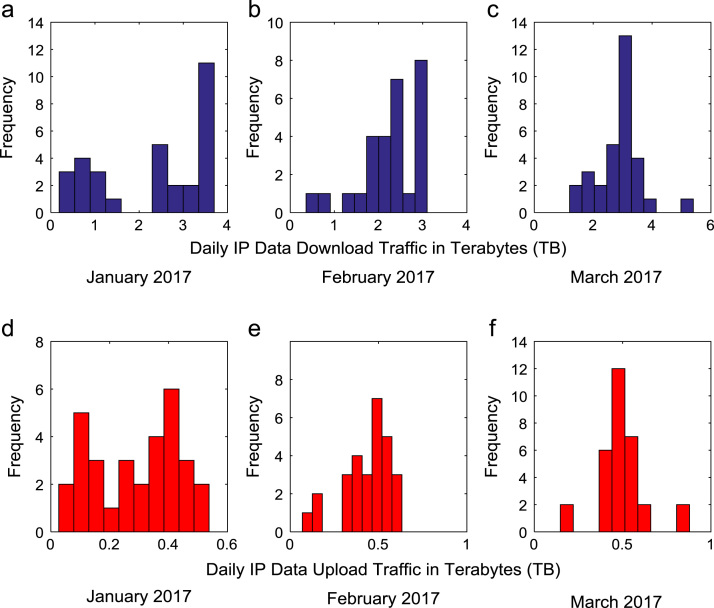
(a)–(f). Frequency distributions of data download and upload traffic in first quarter, 2017.

**Fig. 12:**
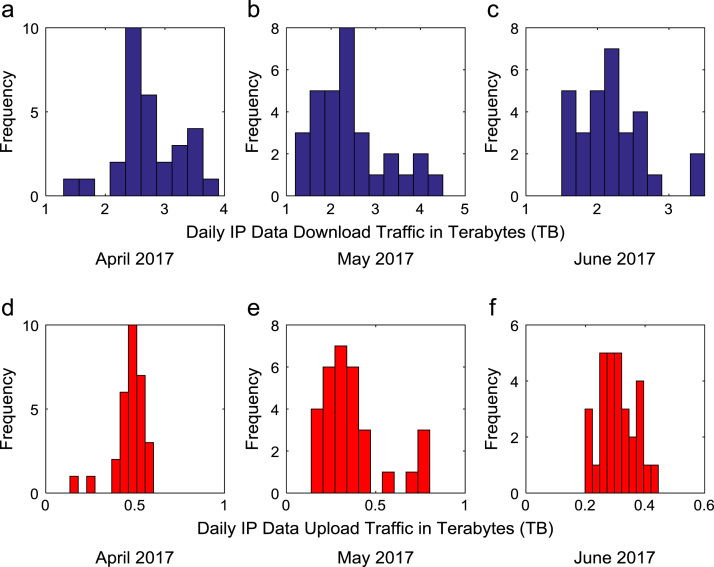
(a)–(f). Frequency distributions of data download and upload traffic in second quarter, 2017.

**Fig. 13:**
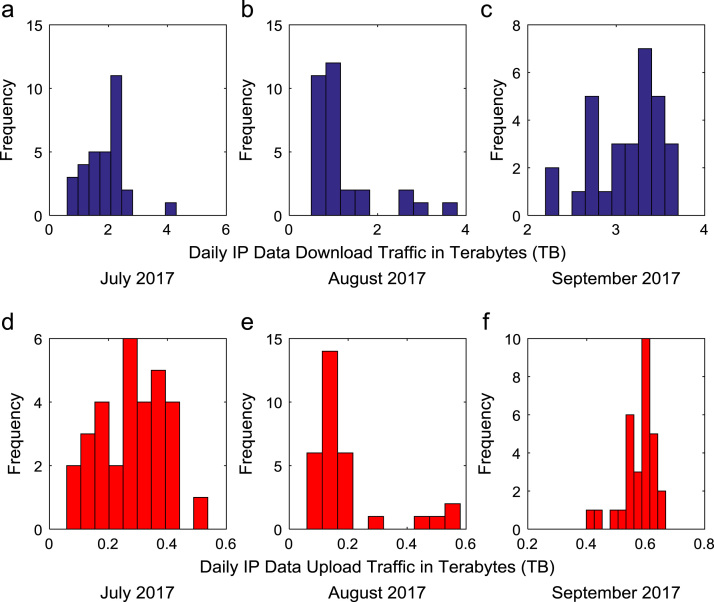
(a)–(f). Frequency distributions of data download and upload traffic in third quarter, 2017.

**Fig. 14:**
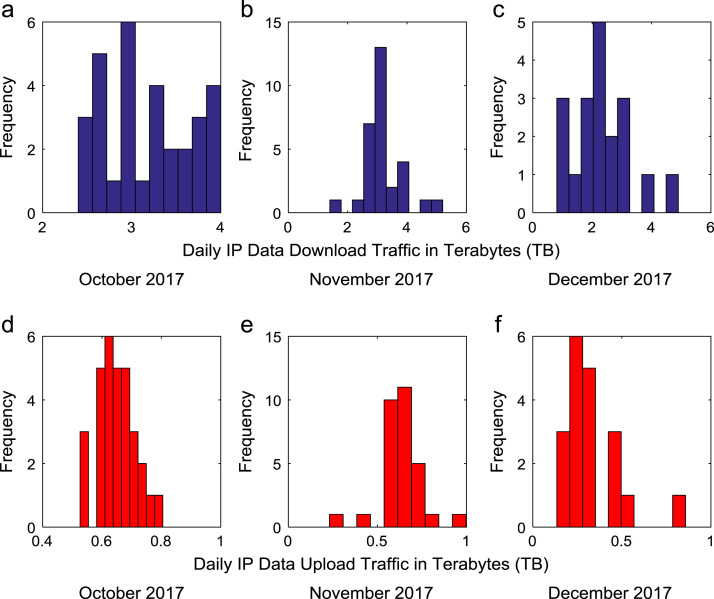
(a)–(f). Frequency distributions of data download and upload traffic in fourth quarter, 2017.

Time series plots are provided to show the trends of data download and upload volume within the smart campus throughout the 12-month period. Fig. 3, Fig. 4, Fig. 5, Fig. 6 show the trends in data download traffic for the first, second, third, and fourth quarters of year 2017 respectively. Similarly, the patterns of data upload traffic for the first, second, third, and fourth quarters of year 2017 are shown in Fig. 7, Fig. 8, Fig. 9, Fig. 10 respectively. Frequency distributions of the dataset are illustrated using histograms. Fig. 11, Fig. 12, Fig. 13, Fig. 14 show the histogram distributions of the data traffic volume for first, second, third, and fourth quarters of 2017.

**Fig. 15:**
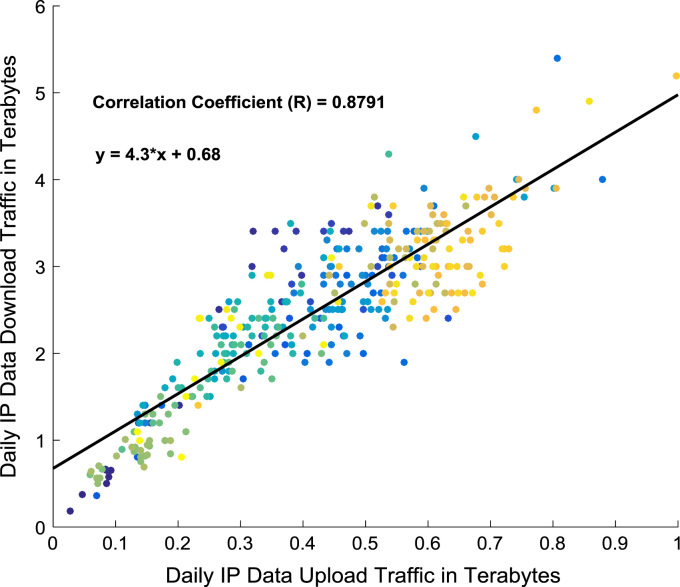
Scatter plot of data download traffic and data upload traffic.

**Fig. 16:**
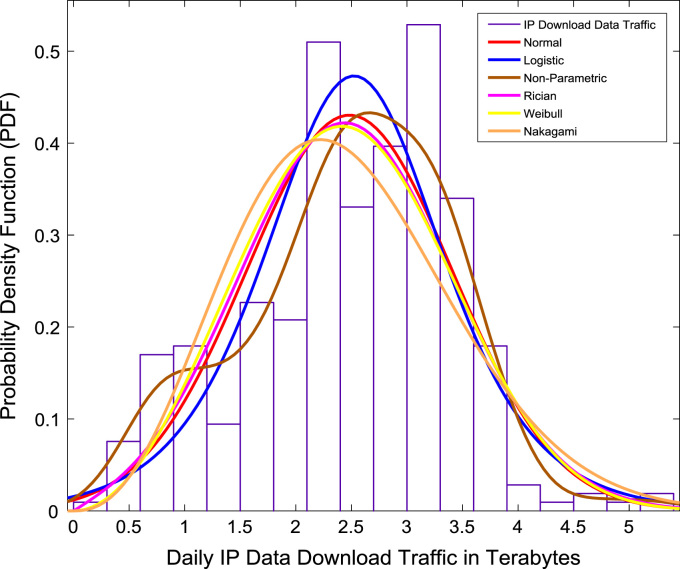
Download data traffic distribution fittings using PDF models.

**Fig. 17:**
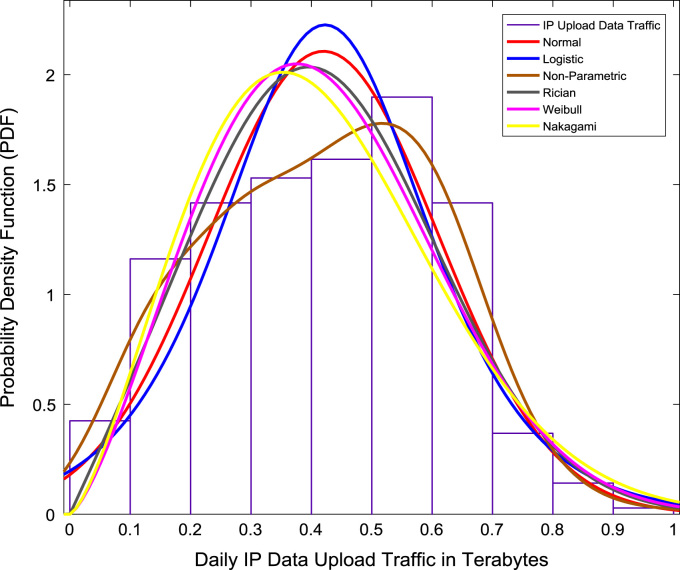
Upload data traffic distribution fittings using PDF models.

**Fig. 18:**
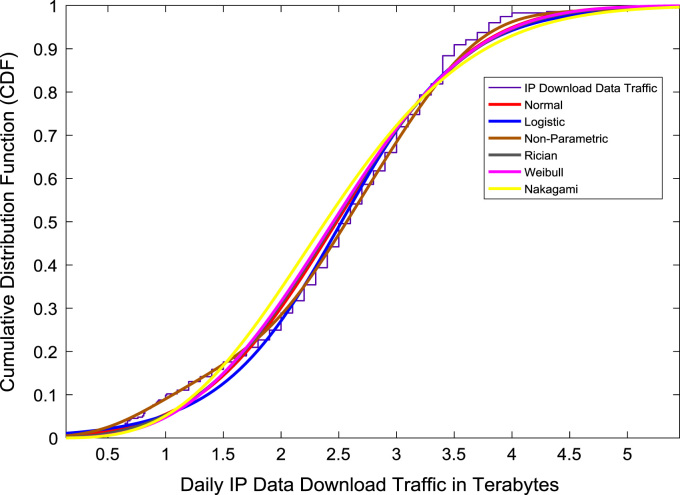
Download data traffic distribution fittings using CDF models.

**Fig. 19:**
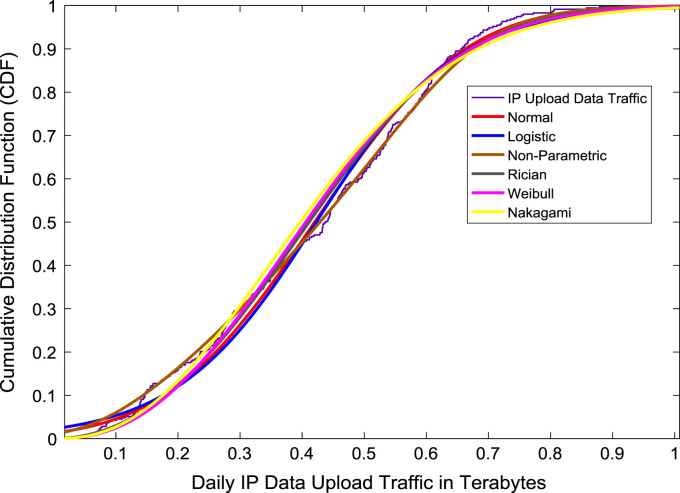
Upload data traffic distribution fittings using CDF models.

Correlation and regression analyses are performed to establish a linear relationship the data download traffic and data upload traffic. The relationship yielded a correlation coefficient (R) of 0.8791. A linear regression equation that represent the relationship is provided in the scatter plot shown in Fig. 15. Probability Density Functions (PDFs) and Cumulative Distribution Functions (CDFs) of the dataset are also computed. PDF and CDF models of Normal, Logistic, Non-parametric, Rician, Weibull and Nakagami distributions were used to fit the empirical data as shown in Figs. 16 and 17. The CDF model distribution fittings of the dataset are shown in Figs. 18 and 19. Distribution fitting parameters for download data traffic (January–December, 2017) based on the six distribution models are presented in Table 3. Estimates and standard errors of download data traffic distribution parameters for the six models are presented in Table 4. Similarly, the distribution fitting parameters for upload data traffic (January–December, 2017) based on the six distribution models are presented in Table 5. Estimates and standard errors of download data traffic distribution parameters for the six models are presented in Table 6.

**Table 3:** Distribution fitting parameters for download data traffic (January–December, 2017).

	**Normal**	**Logistic**	**Rician**	**Weibull**	**Nakagami**
Log Likelihood	−473.562	−477.028	−472.879	−475.457	−485.289
Domain	−∞<y<∞	−∞<y<∞	0<y<∞	0<y<∞	0<y<∞
Mean	2.483	2.524	2.482	2.477	2.462
Variance	0.859	0.919	0.858	0.8313	0.958

**Table 4:** Estimates and standard errors of download data traffic distribution parameters.

	**Normal**		**Logistic**		**Rician**		**Weibull**		**Nakagami**	
**Parameter**	Approx	Std Err	Approx	Std Err	Approx	Std Err	Approx	Std Err	Approx	Std Err
µ	2.483	0.049	2.524	0.049	2.249	0.061	2.776	0.052	1.680	0.116
σ	0.927	0.035	0.528	0.023	0.991	0.043	2.958	0.127	7.020	0.288

**Table 5:** Distribution fitting parameters for upload data traffic (January–December, 2017).

	**Normal**	**Logistic**	**Rician**	**Weibull**	**Nakagami**
Log Likelihood	86.969	75.92	93.032	90.011	86.668
Domain	−∞<y<∞	−∞<y<∞	0<y<∞	0<y<∞	0<y<∞
Mean	0.420	0.423	0.421	0.420	0.417
Variance	0.0359	0.041	0.035	0.035	0.038

**Table 6:** Estimates and standard errors of upload data traffic distribution parameters.

	**Normal**		**Logistic**		**Rician**		**Weibull**		**Nakagami**	
**Parameter**	Approx	Std Err	Approx	Std Err	Approx	Std Err	Approx	Std Err	Approx	Std Err
µ	0.420	0.010	0.422	0.010	0.345	0.017	0.473	0.011	1.211	0.082
σ	0.189	0.007	0.112	0.004	0.216	0.012	2.375	0.104	0.212	0.010

**Fig. 20:**
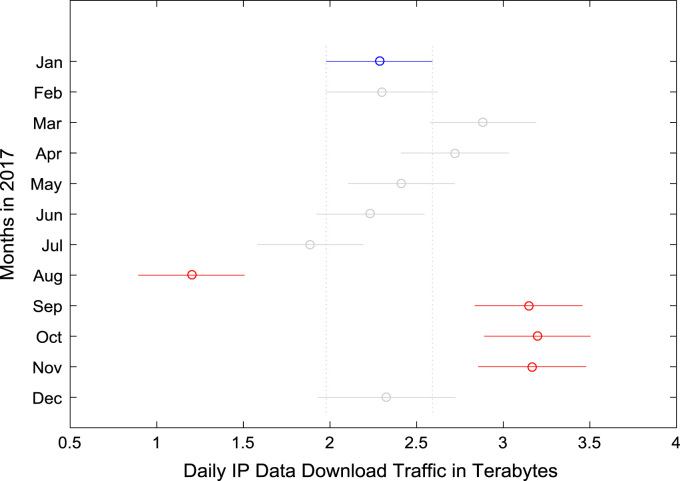
Graphical representation of multiple post-hoc test result for download data traffic.

**Fig. 21:**
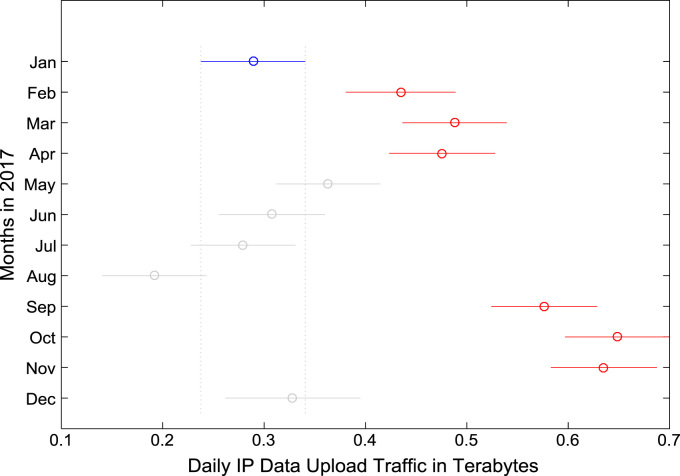
Graphical representation of multiple post-hoc test result for download data traffic.

Furthermore, Analysis of Variance (ANOVA) and multiple post-hoc tests are conducted to understand the statistical difference(s) in the Internet traffic volume, if any, across the 12-month period. The results of the ANOVA test and the multiple post-hoc test conducted on download data traffic are presented in Tables 7 and 8 respectively. Likewise, the results of the ANOVA test and the multiple post-hoc test conducted on upload data traffic are presented in Tables 9 and 10 respectively. The multiple post-hoc comparison results for download data traffic and upload data traffic are depicted graphically in Figs. 20 and 21.

**Table 7:** ANOVA test results for download data traffic.

**Source of Variation**	**Sum of Squares**	**Degree of Freedom**	**Mean Squares**	**F Statistic**	**Prob>F**
Columns	116.44	11	10.59	19.41	3.16×10^−30^
Error	185.93	341	0.55		
Total	302.37	352

**Table 8:** Multiple post-hoc test results for download data traffic.

**Groups Compared**		**Lower limits for 95% confidence intervals**	**Mean Difference**	**Upper limits for 95% confidence intervals**	***p*-value**
Jan	Feb	−0.6435	−0.0144	0.6148	1.0000
Jan	Mar	−1.2126	−0.5997	0.0133	0.0619
Jan	Apr	−1.0538	−0.4358	0.1822	0.4732
Jan	May	−0.7416	−0.1287	0.4842	0.9999
Jan	Jun	−0.5672	0.0509	0.6689	1.0000
Jan	Jul	−0.2145	0.3984	1.0113	0.6053
Jan	Aug	0.4713	1.0842	1.6971	0.0000
Jan	Sep	−1.4805	−0.8625	−0.2445	0.0003
Jan	Oct	−1.5255	−0.9126	−0.2996	0.0001
Jan	Nov	−1.5005	−0.8825	−0.2645	0.0002
Jan	Dec	−0.7457	−0.0426	0.6604	1.0000
Feb	Mar	−1.2144	−0.5853	0.0438	0.0971
Feb	Apr	−1.0555	−0.4214	0.2127	0.5702
Feb	May	−0.7435	−0.1143	0.5148	1.0000
Feb	Jun	−0.5689	0.0652	0.6993	1.0000
Feb	Jul	−0.2164	0.4128	1.0419	0.5907
Feb	Aug	0.4694	1.0986	1.7277	0.0000
Feb	Sep	−1.4822	−0.8481	−0.2140	0.0008
Feb	Oct	−1.5273	−0.8982	−0.2691	0.0002
Feb	Nov	−1.5022	−0.8681	−0.2340	0.0005
Feb	Dec	−0.7455	−0.0283	0.6890	1.0000
Mar	Apr	−0.4541	0.1639	0.7819	0.9994
Mar	May	−0.1420	0.4710	1.0839	0.3327
Mar	Jun	0.0325	0.6505	1.2686	0.0288
Mar	Jul	0.3851	0.9981	1.6110	0.0000
Mar	Aug	1.0709	1.6839	2.2968	0.0000
Mar	Sep	−0.8808	−0.2628	0.3552	0.9658
Mar	Oct	−0.9258	−0.3129	0.3000	0.8827
Mar	Nov	−0.9008	−0.2828	0.3352	0.9423
Mar	Dec	−0.1461	0.5570	1.2601	0.2857
Apr	May	−0.3109	0.3071	0.9251	0.9007
Apr	Jun	−0.1364	0.4867	1.1097	0.3072
Apr	Jul	0.2162	0.8342	1.4522	0.0006
Apr	Aug	0.9020	1.5200	2.1380	0.0000
Apr	Sep	−1.0497	−0.4267	0.1964	0.5217
Apr	Oct	−1.0948	−0.4768	0.1412	0.3264
Apr	Nov	−1.0697	−0.4467	0.1764	0.4458
Apr	Dec	−0.3144	0.3932	1.1007	0.8096
May	Jun	−0.4384	0.1796	0.7976	0.9986
May	Jul	−0.0858	0.5271	1.1400	0.1754
May	Aug	0.6000	1.2129	1.8258	0.0000
May	Sep	−1.3518	−0.7338	−0.1157	0.0059
May	Oct	−1.3968	−0.7839	−0.1709	0.0017
May	Nov	−1.3718	−0.7538	−0.1357	0.0039
May	Dec	−0.6170	0.0861	0.7891	1.0000
Jun	Jul	−0.2705	0.3475	0.9655	0.7972
Jun	Aug	0.4153	1.0333	1.6514	0.0000
Jun	Sep	−1.5364	−0.9133	−0.2903	0.0001
Jun	Oct	−1.5815	−0.9634	−0.3454	0.0000
Jun	Nov	−1.5564	−0.9333	−0.3103	0.0001
Jun	Dec	−0.8010	−0.0935	0.6140	1.0000
Jul	Aug	0.0729	0.6858	1.2987	0.0136
Jul	Sep	−1.8789	−1.2609	−0.6428	0.0000
Jul	Oct	−1.9239	−1.3110	−0.6980	0.0000
Jul	Nov	−1.8989	−1.2809	−0.6628	0.0000
Jul	Dec	−1.1441	−0.4410	0.2620	0.6587
Aug	Sep	−2.5647	−1.9467	−1.3286	0.0000
Aug	Oct	−2.6097	−1.9968	−1.3838	0.0000
Aug	Nov	−2.5847	−1.9667	−1.3486	0.0000
Aug	Dec	−1.8299	−1.1268	−0.4238	0.0000
Sep	Oct	−0.6681	−0.0501	0.5679	1.0000
Sep	Nov	−0.6431	−0.0200	0.6031	1.0000
Sep	Dec	0.1123	0.8198	1.5273	0.0084
Oct	Nov	−0.5879	0.0301	0.6481	1.0000
Oct	Dec	0.1669	0.8699	1.5730	0.0031
Nov	Dec	0.1323	0.8398	1.5473	0.0059

**Table 9:** ANOVA test results for upload data traffic.

**Source of Variation**	**Sum of Squares**	**Degree of Freedom**	**Mean Squares**	**F Statistic**	**Prob > F**
Columns	7.38	11	0.67	43.58	2.03 × 10^−58^
Error	5.25	341	0.02		
Total	12.63	352

**Table 10:** Multiple post-hoc test results for upload data traffic.

**Groups Compared**		**Lower limits for 95% confidence intervals**	**Mean Difference**	**Upper limits for 95% confidence intervals**	***p*-value**
Jan	Feb	−0.2513	−0.1456	−0.0399	0.0004
Jan	Mar	−0.3018	−0.1988	−0.0958	0.0000
Jan	Apr	−0.2904	−0.1866	−0.0827	0.0000
Jan	May	−0.1771	−0.0741	0.0289	0.4391
Jan	Jun	−0.1224	−0.0186	0.0852	1.0000
Jan	Jul	−0.0933	0.0097	0.1126	1.0000
Jan	Aug	−0.0056	0.0974	0.2004	0.0843
Jan	Sep	−0.3911	−0.2873	−0.1835	0.0000
Jan	Oct	−0.4622	−0.3593	−0.2563	0.0000
Jan	Nov	−0.4499	−0.3461	−0.2422	0.0000
Jan	Dec	−0.1573	−0.0392	0.0789	0.9953
Feb	Mar	−0.1589	−0.0532	0.0525	0.8930
Feb	Apr	−0.1474	−0.0409	0.0656	0.9843
Feb	May	−0.0342	0.0715	0.1772	0.5413
Feb	Jun	0.0205	0.1270	0.2336	0.0055
Feb	Jul	0.0496	0.1553	0.2610	0.0001
Feb	Aug	0.1374	0.2431	0.3488	0.0000
Feb	Sep	−0.2482	−0.1416	−0.0351	0.0009
Feb	Oct	−0.3193	−0.2136	−0.1079	0.0000
Feb	Nov	−0.3070	−0.2004	−0.0939	0.0000
Feb	Dec	−0.0141	0.1064	0.2269	0.1456
Mar	Apr	−0.0916	0.0122	0.1161	1.0000
Mar	May	0.0217	0.1247	0.2277	0.0044
Mar	Jun	0.0764	0.1802	0.2840	0.0000
Mar	Jul	0.1055	0.2085	0.3114	0.0000
Mar	Aug	0.1932	0.2962	0.3992	0.0000
Mar	Sep	−0.1923	−0.0885	0.0153	0.1862
Mar	Oct	−0.2634	−0.1605	−0.0575	0.0000
Mar	Nov	−0.2511	−0.1473	−0.0434	0.0002
Mar	Dec	0.0415	0.1596	0.2777	0.0006
Apr	May	0.0086	0.1124	0.2163	0.0206
Apr	Jun	0.0633	0.1680	0.2726	0.0000
Apr	Jul	0.0924	0.1962	0.3000	0.0000
Apr	Aug	0.1801	0.2840	0.3878	0.0000
Apr	Sep	−0.2054	−0.1007	0.0039	0.0723
Apr	Oct	−0.2765	−0.1727	−0.0689	0.0000
Apr	Nov	−0.2642	−0.1595	−0.0548	0.0000
Apr	Dec	0.0285	0.1473	0.2662	0.0030
May	Jun	−0.0483	0.0555	0.1594	0.8460
May	Jul	−0.0192	0.0838	0.1868	0.2473
May	Aug	0.0686	0.1715	0.2745	0.0000
May	Sep	−0.3170	−0.2132	−0.1093	0.0000
May	Oct	−0.3881	−0.2851	−0.1822	0.0000
May	Nov	−0.3758	−0.2719	−0.1681	0.0000
May	Dec	−0.0832	0.0349	0.1530	0.9983
Jun	Jul	−0.0756	0.0283	0.1321	0.9992
Jun	Aug	0.0122	0.1160	0.2198	0.0139
Jun	Sep	−0.3734	−0.2687	−0.1640	0.0000
Jun	Oct	−0.4445	−0.3407	−0.2368	0.0000
Jun	Nov	−0.4321	−0.3275	−0.2228	0.0000
Jun	Dec	−0.1395	−0.0206	0.0983	1.0000
Jul	Aug	−0.0152	0.0878	0.1907	0.1862
Jul	Sep	−0.4008	−0.2969	−0.1931	0.0000
Jul	Oct	−0.4719	−0.3689	−0.2659	0.0000
Jul	Nov	−0.4596	−0.3557	−0.2519	0.0000
Jul	Dec	−0.1670	−0.0489	0.0693	0.9721
Aug	Sep	−0.4885	−0.3847	−0.2809	0.0000
Aug	Oct	−0.5597	−0.4567	−0.3537	0.0000
Aug	Nov	−0.5473	−0.4435	−0.3396	0.0000
Aug	Dec	−0.2548	−0.1366	−0.0185	0.0086
Sep	Oct	−0.1758	−0.0720	0.0319	0.5017
Sep	Nov	−0.1635	−0.0588	0.0459	0.7988
Sep	Dec	0.1292	0.2481	0.3669	0.0000
Oct	Nov	−0.0906	0.0132	0.1170	1.0000
Oct	Dec	0.2019	0.3200	0.4382	0.0000
Nov	Dec	0.1880	0.3069	0.4257	0.0000
